# Work-related musculoskeletal disorders and digitalization: past adoption, current utilization, and future concerns

**DOI:** 10.1186/s12889-025-23466-w

**Published:** 2025-07-03

**Authors:** Young-Mee Kim, Sung-il Cho

**Affiliations:** Department of Public Health Science, Graduate School of Public Health and Institute of Health and Environment, Seoul National University, 1 Gwanak-ro, Gwanak‐gu, Seoul, 08826 South Korea

**Keywords:** Digitalization, Automation, Musculoskeletal disorders, Resilience, COVID-19, South Korea

## Abstract

**Background:**

The COVID-19 pandemic rapidly accelerated workplace digitalization, significantly heightening concerns about its impact. While the physical risks associated with digitalization, particularly musculoskeletal disorders (MSDs), have been widely studied, research on psychosocial factors—especially concerns about future digitalization—remains relatively limited. This study aimed to assess whether concerns about future digitalization are associated with MSDs, while considering past adoption and current utilization of digital technologies. Additionally, it examined the moderating effects of various factors, including socioeconomic and work-related variables, on this relationship.

**Methods:**

Data from the 2020 Korean Working Conditions Survey (KWCS), which included a representative sample of 48,604 workers in South Korea, were analyzed. A multiple logistic regression analysis was conducted to explore the association between concerns about future digitalization and MSDs. The model controlled for past adoption and current utilization of digitalization, as well as other relevant covariates. Furthermore, interaction terms with moderating factors were incorporated to investigate potential moderating effects within the model.

**Results:**

Concerns about future digitalization were significantly associated with the likelihood of MSDs, even after accounting for past adoption and current utilization (OR: 1.08, 95% CI: 1.04–1.14). The impact of these concerns on MSDs was moderated by education level, with higher education levels mitigating the effect (OR: 1.41 for below middle school, 0.85 for high school or college, and 0.71 for university or above).

**Conclusions:**

Recognizing concerns related to future digitalization as a critical psychosocial risk factor is essential, as they influence the development of MSDs. Future research should focus on alleviating these concerns and enhancing workers’ resilience in increasingly digitalized work environments. These findings underscore the importance for policymakers and employers to incorporate psychosocial considerations into digital transition strategies and to provide targeted support for vulnerable worker populations.

**Supplementary Information:**

The online version contains supplementary material available at 10.1186/s12889-025-23466-w.

## Introduction

The rapid advancement of technologies is transforming work across both blue- and white-collar sectors [1]. While digitalization has improved productivity and safety by reducing manual labor and hazardous exposures [2–4], it also raises concerns about job loss and wage decline [5–7]. One study, for example, found that one additional robot per 1,000 workers reduces employment by 0.2% and wages by 0.42% [6]. Beyond its economic consequences, workplace digitalization also has significant implications for worker health. Recent studies suggest that digital work environments—particularly those shaped by remote work, algorithmic management, and constant connectivity—have transformed how work is organized and intensified job demands, leading to negative strain reactions such as information overload, fatigue, and emotional exhaustion [8, 9]. Moreover, digital surveillance and the normalization of constant availability reduce employees’ sense of control and recovery opportunities, thereby exacerbating psychological strain [8]. In addition, sedentary work, repetitive computer use, and low task variety have been linked to musculoskeletal disorders (MSDs) [10].

MSDs are intrinsically linked to occupational activities and are among the most prevalent occupational diseases in modern work environments [11–13]. They are multifactorial, influenced by both individual characteristics (e.g., age, sex, genetic predisposition) and job-related conditions [14, 15]. Notably, psychosocial factors have been identified as significant contributors to MSDs, with proposed biomechanical and physiological pathways [16–18]. For example, job stress and work–life conflict—such as role conflict between home and work—can elevate psychological tension, leading to increased muscle activity, poor posture, or maladaptive work habits, thereby increasing the risk of MSDs, especially in the neck, shoulders, and lower back [19–21]. Laboratory studies support this, showing that psychosocial stress is associated with heightened muscle activation [22, 23].

While research on the physical risks of digitalization for musculoskeletal health is growing, there remains a notable lack of studies specifically examining the relationship between digitalization-related psychological risk factors and MSDs. In addition, although existing research has examined various psychological aspects of digitalization, most studies have primarily focused on technostress, job insecurity, and psychological burdens such as techno-overload and complexity, all of which have been shown to negatively affect mental health [24–26]. These studies tend to emphasize current psychological stressors experienced in digital work environments, while paying limited attention to how workers’ future-oriented concerns—particularly their anticipatory anxiety and stress about the long-term implications of digital transformation—may influence health outcomes such as MSDs.

To our knowledge, there is a lack of empirical studies that directly link future-oriented concerns about digitalization with MSDs, despite the relevance of such concerns in the context of accelerating structural changes in the labor market. One recent study has examined the association between future-oriented concerns and sleep disturbances, but we are not aware of any research that has investigated MSDs as the outcome [27]. This gap is particularly notable given the increasing importance of these concerns in today’s rapidly evolving labor landscape driven by digitalization. *Future concerns about digitalization* refer to workers’ anticipatory anxiety and stress regarding the potential negative impacts of technological advancements on the future of work, employment stability, job autonomy, and social inequality [27]. This concept is distinct from short-term job insecurity, as it reflects long-term and uncertain threats stemming from structural transformation. These concerns have gained particular relevance following the rapid acceleration of digitalization during the COVID-19 pandemic, as they are closely tied to uncertainties about job security, autonomy, and evolving work structures. Unlike short-term job insecurity, they represent deeper anxieties about long-term structural changes in the labor market.

South Korea’s advanced Information and Communication Technology (ICT) infrastructure and rapid digital adoption make it an important context for examining how digitalization affects worker health [28]. The COVID-19 pandemic further accelerated this digital shift, fundamentally altering workplace dynamics and expanding the use of digital tools and remote work practices [29]. In this context, the Korea Working Conditions Survey (KWCS), conducted every three years, provides a valuable source of demographic and occupational data [30]. Its sixth wave, carried out from October 2020 to April 2021 at the height of the pandemic, offers timely and relevant data for analyzing the health effects of COVID-19–driven workplace digitalization [31].

Using this dataset, the present study examined the association between concerns about future digitalization and MSDs, while accounting for contextual factors such as prior adoption and current use of digital technologies. It also explored whether this association is moderated by socioeconomic and occupational characteristics. Specifically, the study addressed two key research questions: [1] Are concerns about future digitalization associated with MSDs [2]? Which socio-demographic and work-related factors moderate this relationship? By focusing on future-oriented concerns—a psychosocial factor that has received limited attention in research on MSDs—this study highlights an underexplored dimension of workplace health. These findings help identify emerging health risks in digitalized workplaces and support efforts to improve working conditions.

## Methods

### Data source

The data for this study were obtained from the 6th KWCS KWCS, a cross-sectional survey conducted in 2020 using a nationally representative sample of South Korea’s workforce [30]. The 6th KWCS questionnaire was adapted from the master version of the 7th European Working Conditions Survey (EWCS), officially provided for translation [32]. The final Korean version was developed through expert consultation and further refined based on feedback from the Ministry of Employment and Labor and Statistics Korea. All variables used in the analysis—including past adoption, current utilization, and concerns about future digitalization—were derived from items included in this revised questionnaire. The survey targeted individuals aged 15 and older who were actively participating in the labor force. The 2020 KWCS employed a stratified cluster sampling method based on proportional probability to obtain a nationally representative sample [32]. A total of 50,538 respondents completed the structured questionnaire.

### Study sample

Of the total respondents, 1,934 individuals with missing values for key variables were excluded, resulting in a final analytic sample of 48,604 participants, representing 96.2% of the original dataset. Data collection was conducted primarily through face-to-face interviews, with trained interviewers visiting respondents’ homes. Approximately 60% of responses were gathered using tablet-assisted personal interviewing (TAPI), while the remaining 40% were collected using pen-and-paper interviewing (PAPI) when TAPI was not feasible [33]. The questionnaire covered a wide range of workplace hazards, including physical, biological, and psychological hazards, as well as inappropriate ergonomic conditions. The reliability and validity of the KWCS data have been well documented [34].

### Measures

**Fig. 1:**
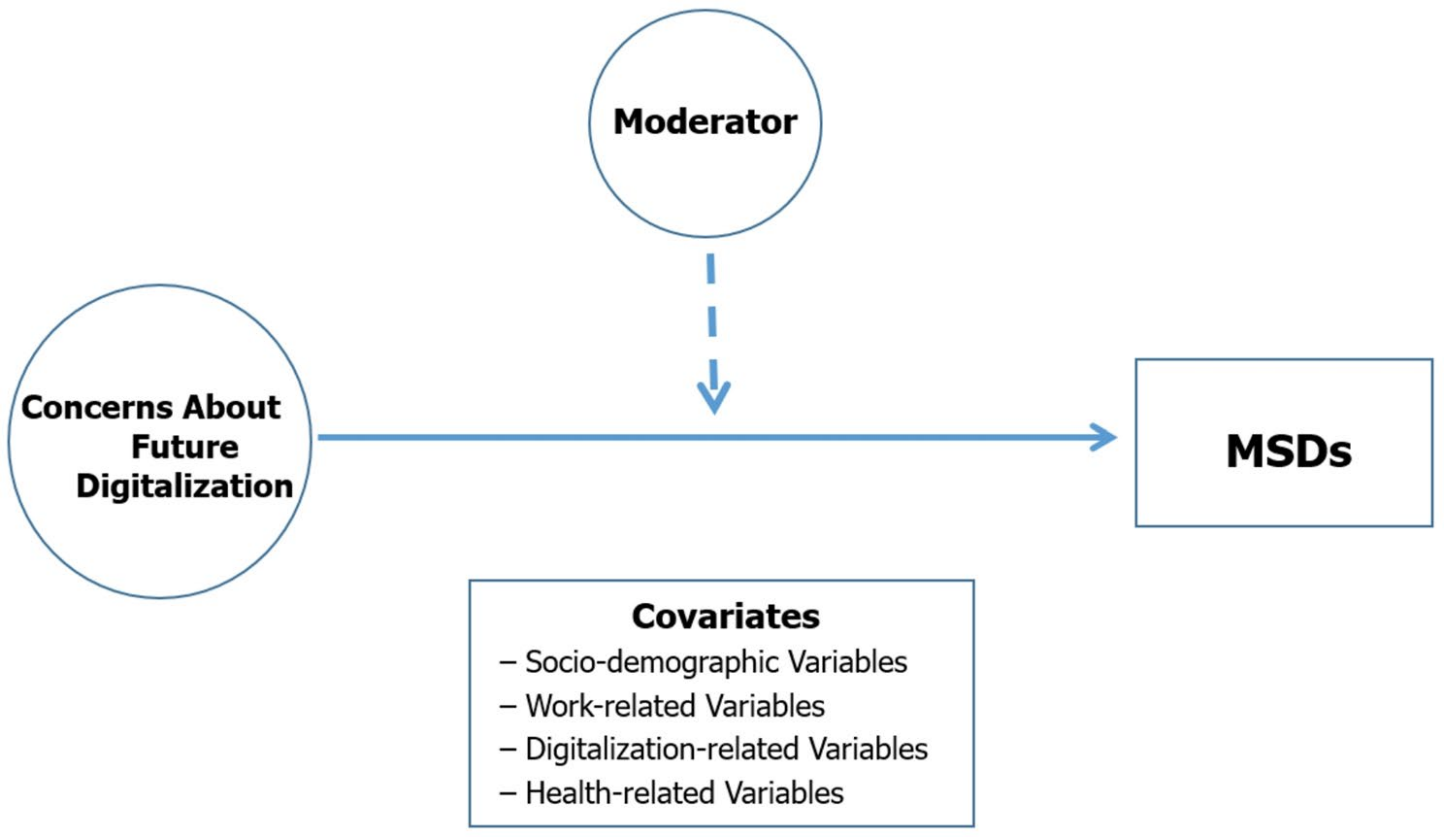
Conceptual model illustrating the association between concerns about future digitalization and MSDs, including covariates and a potential moderator

To clarify the overall analytical framework of this study, we first present a conceptual model. Figure 1 illustrates the hypothesized relationship between concerns about future digitalization and MSDs, along with covariates and potential moderating factors.

#### Digitalization at work

##### Past digitalization adoption

Past digitalization adoption was assessed using the question: “During the last 3 years, have any of the following been introduced at your company or organization?” The specific items were: (a) the introduction or significant change of new information and communication devices, and (b) the introduction or significant change in new work methods. Each question required a “yes” or “no” response. Participants who answered “yes” to at least one of these items were considered to have had relevant experience with digital technologies.

##### Current digitalization utilization

Current digitalization utilization in the work environment was measured with the question: “How often did you use the following methods while working?” The question covered three specific aspects: (a) using computers, laptops, tablets, or smartphones for work, (b) using other devices (equipment or machinery) for internet access or operation, and (c) performing tasks assigned automatically without human intervention. Responses of “all the time” or “almost all the time” were classified as “yes,” while responses of “three-quarters of working time,” “half of working time,” “one-quarter of working time,” “almost none,” and “never” were classified as “no.” Participants who answered “yes” to at least one of the three items were considered to be in the group utilizing digitalization in their current work environment.

##### Engagement in remote work

Engagement in remote work was measured by the question: “In the past year, how often did you work from home?” Responses of “always,” “almost always,” “sometimes,” and “occasionally” were classified as remote work, while the response “never” was classified as not engaging in remote work.

##### Concerns about future digitalization

Concerns about future digitalization were investigated by asking participants: “How worried were you about these situations affecting your work?” The specific concerns included: (a) future job changes that may reduce control over how the job is done, (b) future changes that may make it harder to use their skills, (c) potential reductions in pay, (d) being transferred to a less interesting job, and (e) unexpected changes in work hours. Responses were measured on a scale of “very worried,” “fairly worried,” “not very worried,” and “not at all worried.” For each concern, responses of “very worried” and “fairly worried” were classified as having concerns, while responses of “not very worried” and “not at all worried” were classified as not having concerns. The five items assessing concerns about future digitalization demonstrated good internal consistency (Cronbach’s alpha = 0.84). Participants were classified as having concerns about future digitalization if they expressed concern for at least one of the five items.

#### MSDs

The presence of MSDs was assessed using the question: “In the past 12 months, have you experienced any of the following health issues?” This question addressed three specific areas: (a) back pain, (b) muscle pain in the shoulders, neck, and/or upper limbs, and (c) muscle pain in the lower limbs (hips, legs, knees, feet, etc.). Participants could respond with either “yes” or “no.” Those who answered “yes” to at least one of the three items were classified as having MSDs.

#### Covariates

##### Socio-demographic variables

The socio-demographic characteristics encompassed age groups (15–49, 50–59, and 60+), gender (male and female), family structure (single and non-single), education level (middle school or below, high school or college, and university or higher), and salary brackets (less than $1,500, $1,500 to $2,299, $2,300 to $2,999, $3,000 and above, and no answer).

##### Work-related variables

We also selected work-related variables that have been reported to be linked to MSDs [15]. Employment type was categorized into three groups: self-employed, employee, and employs others. Occupation was classified into four categories: white-collar, sales and service, skilled blue-collar, and unskilled and other (including non-skilled, agriculture, or forestry). Shift work was divided into non-shift and shift, and working hours were categorized as < 40 h/week, 40–54 h/week, and ≥ 55 h/week. Perceived job stress was assessed with the statement: “I experience stress at work.” Responses were classified into two groups: high perceived job stress (always or most of the time) and low perceived job stress (sometimes, rarely, or never).

Physical demands at work were evaluated using five questions regarding: (a) the amount of time spent in exhausting or uncomfortable positions, (b) lifting or moving individuals, (c) carrying or moving heavy objects, (d) prolonged standing, and (e) repetitive movements of the hands or arms. Responses were measured on a 7-point scale ranging from 1 (never) to 7 (all the time). Participants were categorized into high or low physical demand groups based on the median score. Job insecurity was measured with two statements: (a) “I might lose my job in the next 6 months,” with “strongly agree” and “agree” categorized as “yes”; and (b) “If I were to lose or quit my current job, it would be easy for me to find a job with a similar salary,” with “disagree” and “strongly disagree” categorized as “yes.” Participants who responded “yes” to at least one of these two statements were classified into the high job insecurity group.

##### Health-related variables

Health-related indicators were assessed using two measures: self-rated health (SRH) and the presence of chronic health conditions. SRH was evaluated by asking participants: “How would you rate your overall health?” Responses were grouped into three categories: good (very good or good), average (fair), and poor (poor or very poor). Chronic health conditions were identified with the question: “Do you have any illness or health problem that has lasted, or is expected to last, for more than six months?” Responses were either “yes” or “no.”

### Statistical methods

We first conducted chi-square tests to examine whether the distribution of MSDs differed significantly across socio-demographic, work-related, and digitalization-related variables. This test was selected due to its appropriateness for assessing associations between categorical variables. A *p*-value of less than 0.05 was considered indicative of statistical significance. Because the outcome variable—the presence or absence of MSDs—is binary, we used multivariable logistic regression analyses to assess the independent association between concerns about future digitalization and MSDs, adjusting for potential confounders including past adoption and current utilization of digitalization, as well as age, sex, household type, education level, income, employment type, occupational category, shift type, weekly work hours, job stress, physical demands, job insecurity, SRH, and chronic health conditions. Before model estimation, we assessed multicollinearity using the generalized variance inflation factor (GVIF), calculated with the R function vif from the *car* package [35]. Adjusted GVIF values for all variables were below 2.0, indicating no multicollinearity concern. Results are reported as odds ratios (ORs) with 95% confidence intervals (CIs). An OR greater than 1 indicates increased odds of MSDs, while an OR less than 1 indicates decreased odds. Associations are considered statistically significant if the 95% CI does not include 1. Additionally, interaction terms involving concerns about future digitalization and key covariates (age, gender, education level, and occupational category) have been included to assess potential moderating effects on the relationship between digitalization concerns and MSDs. All analyses were conducted using R software (version 4.2).

## Results

### Prevalence of MSDs according to socio-demographic, work, and digitalization characteristics

Table 1 summarizes the characteristics of the study population and the distribution of MSDs according to socio-demographic, work-related, and digitalization-related factors. Group differences in categorical variables were assessed using chi-square tests, with statistical significance set at *p* < 0.05. Among the 48,604 participants, 45.1% reported MSDs. Prevalence was higher in women (49.9%) than in men (39.8%) (*p* < 0.001). It was also higher among those with less than a middle school education (74.1%) compared to university graduates (30.4%) (*p* < 0.001). Participants with high job stress reported more MSDs (47.8%) than those without (44.0%). Similarly, those with high physical demands showed greater prevalence (57.1%) than those with low physical demands (34.0%) (*p* < 0.001).

**Table 1:** Participant characteristics and prevalence of MSDs according to socio-demographic, Work-Related, and Digitalization-Related factors (*n* = 48,604)

		Non-MSDs *n* = 26,728 (54.9)	MSDs *n* = 21,975 (45.1)	*p* value ^a^
**Socio-demographic variables**				
**Age**				
15–29 years	4,664 (9.6)	3,631 (77.9)	1,033 (22.1)	< 0.001
30–39 years	8,369 (17.2)	5,803 (69.3)	2,566 (30.7)	
40–49 years	10,676 (22.0)	6,482 (60.7)	4,194 (39.3)	
50–59 years	12,100 (24.9)	6,237 (51.5)	5,863 (48.5)	
60 years and older	12,795 (26.3)	4,520 (35.3)	8,275 (64.7)	
**Sex**				
Men	22,832 (47.0)	13,754 (60.2)	9,078 (39.8)	< 0.001
Women	25,772 (53.0)	12,919 (50.1)	12,853 (49.9)	
**Household type**				
Single	10,526 (21.7)	5,558 (52.8)	4,968 (47.2)	< 0.001
Non-Single	38,078 (78.3)	21,115 (55.5)	16,963 (44.5)	
**Education level**				
Below middle school	8,050 (16.6)	2,085 (25.9)	5,965 (74.1)	< 0.001
High school or College	25,963 (53.4)	14,429 (55.6)	11,534 (44.4)	
University or above	14,591 (30.0)	10,159 (69.6)	4,432 (30.4)	
**Salary (USD** ^**b**^ **per month)**				
< 1,500	15,622 (32.2)	6,937 (44.4)	8,685 (55.6)	< 0.001
1,500-2,299	14,393 (29.6)	8,315 (57.8)	6,078 (42.2)	
2,300-2,999	9,325 (19.2)	5,684 (61.0)	2,193 (23.7)	
≥ 3,000	7,138 (14.7)	4,481 (62.8)	1,681 (23.7)	
No-answer	2,126 (4.3)	1,256 (59.1)	870 (40.9)	
**Work-related variables**				
**Employment type**				
Self-employed	15,068 (31.0)	6,952 (46.1)	8,116 (53.9)	< 0.001
Employee	32,013 (65.9)	19,201 (60.0)	12,812 (40.0)	
Employs others	1,523 (3.1)	520 (34.1)	1,003 (65.9)	
**Occupational category**				
White collar	16,265 (33.5)	11,388 (70.0)	4,877 (30.0)	< 0.001
Sales and services	14,728 (30.3)	8,172 (55.5)	6,556 (44.5)	
Skilled blue-collar	12,020 (24.7)	4,772 (39.7)	7,248 (60.3)	
Unskilled and others	5,591 (11.5)	2,341 (41.9)	3,250 (58.1)	
**Shift type**				
Shift	3,492 (7.2)	2,034 (58.2)	1,458 (41.8)	< 0.001
Non-shift	45,112 (92.8)	24,639 (54.6)	20,473 (45.4)	
**Work hours (per week)**				
< 40	11,485 (23.6)	5,206 (45.3)	6,279 (54.7)	< 0.001
40–54	30,586 (62.9)	18,536 (60.6)	12,050 (39.4)	
≥ 55	6,533 (13.4)	2,931 (44.9)	3,602 (55.1)	
**Job stress**				
No	34,378 (70.7)	19,244 (56.0)	15,134 (44.0)	< 0.001
Yes	14,226 (29.3)	7,429 (52.2)	6,797 (47.8)	
**Physical demands**				
Low	25,167 (51.8)	16,607 (66.0)	8,560 (34.0)	< 0.001
High	23,437 (48.2)	10,066 (42.9)	13,371 (57.1)	
**Job insecurity**				
Low	25,814 (53.1)	14,788 (57.3)	11,026 (42.7)	< 0.001
High	22,790 (46.9)	11,885 (52.2)	10,905 (47.8)	
**Digitalization at Work**				
**Past Adoption**				
No	42,872 (88.2)	23,650 (55.2)	19,222 (44.8)	< 0.001
Yes	5,732 (11.8)	3,023 (52.7)	2,709 (47.3)	
**Current utilization**				
No	35,520 (73.1)	18,099 (51.0)	17,421 (49.0)	< 0.001
Yes	13,084 (26.9)	8,574 (65.5)	4,510 (34.5)	
**Remote work**				
No	44,139 (90.8)	24,753 (56.1)	19,386 (43.9)	< 0.001
Yes	4,465 (9.2)	1,920 (43.0)	2,545 (57.0)	
**Concerns about the future**				
No	14,622 (30.1)	8,546 (58.4)	6,076 (41.6)	< 0.001
Yes	33,982 (69.9)	18,127 (53.3)	15,855 (46.7)	
**Health-related variables**				
**Self-rated health**				
Good	32,098 (66.0)	21,450 (66.8)	10,648 (33.2)	< 0.001
Average	13,686 (28.2)	4,936 (31.6)	8,750 (63.9)	
Bad	2,820 (5.8)	287 (10.2)	2,533 (89.8)	
**Chronic health conditions**				
No	43,225 (88.9)	25,924 (60.0)	17,301 (40.0)	< 0.001
Yes	5,379 (11.1)	749 (13.9)	4,630 (86.1)	

Note. Percentages in the shaded areas are the column percentages of each variable. (a) p-values are based on chi-square tests comparing participants with and without MSDs across each categorical variable. (b) In the survey year, the exchange rate was 1,000 KRW = 0.87 USD

Regarding digitalization-related factors, 11.8% of participants had adopted digital technologies in the past, 26.9% were currently using them at work, 9.2% were working remotely, and 69.9% expressed concerns about future digitalization. MSDs prevalence was slightly higher among those with past adoption than those without (47.3% vs. 44.8%, *p* < 0.001). In contrast, current users of digital technologies showed a lower prevalence than non-users (34.5% vs. 49.0%, *p* < 0.001). Remote workers had a notably higher prevalence compared to those not working remotely (57.0% vs. 43.9%, *p* < 0.001). Similarly, those concerned about future digitalization reported more MSDs than those without such concerns (46.7% vs. 41.6%, *p* < 0.001).

### Relationship between concerns about future digitalization and MSDs

As shown in Table 2, concerns about future digitalization were significantly associated with MSDs. Individuals who expressed significant concerns had 1.08 times the odds of reporting MSDs compared to those with fewer concerns (OR: 1.08, 95% CI: 1.03–1.13). In addition, past adoption of digitalization was associated with 1.76 times the odds of MSDs (OR: 1.76, 95% CI: 1.63–1.89). Current utilization of digital tools and participation in remote work were also associated with higher odds of MSDs—1.07 times and 1.26 times, respectively (OR: 1.07, 95% CI: 1.02–1.13; OR: 1.26, 95% CI: 1.17–1.36).

**Table 2:** Association between concerns about future digitalization and MSDs (*n* = 48,604)

Model 1	Odds ratios (95% confidence intervals)
**Education level**	
Below middle school	1.00
High school or College	0.66 (0.61–0.71)^‡^
University or above	0.56 (0.51–0.61)^‡^
**Salary (USD** ^**a**^ **per month)**	
< 1,500	1.00
1,500-2,299	1.01 (0.95–1.08)
2,300-2,999	0.96 (0.90–1.03)
≥ 3,000	0.98 (0.91–1.06)
No-answer	0.91 (0.81–1.01)
**Employment type**	
Self-employed	1.00
Employee	0.89 (0.84–0.94)^‡^
Employs others	0.92 (0.81–1.06)
**Occupational category**	
White collar	1.00
Sales and services	1.11 (1.04–1.18)^†^
Skilled blue-collar	1.76 (1.64–1.89)^‡^
Unskilled and others	1.35 (1.24–1.47)^‡^
**Shift type**	
Non-shift	1.00
Shift	1.07 (0.99–1.16)
**Work hours (per week)**	
< 40	1.00
40–54	1.00 (0.94–1.06)
≥ 55	1.36 (1.26–1.48)^‡^
**Job stress**	
Low	1.00
High	1.32 (1.26–1.38)^‡^
**Physical demands**	
Low	1.00
High	2.15 (2.06–2.25)^‡^
**Job insecurity**	
Low	1.00
High	0.98 (0.93–1.05)
**Digitalization at Work**	
**Past Adoption**	
No	1.00
Yes	1.76 (1.63–1.89)^‡^
**Current utilization**	
No	1.00
Yes	1.07 (1.02–1.13)^†^
**Remote work**	
No	1.00
Yes	1.26 (1.17–1.36)^‡^
**Concerns about future digitalization**	
No	1.00
Yes	1.08 (1.03–1.13)^‡^

^†^*p* < 0.05, ^‡^*p* < 0.01. * Odds ratios were adjusted for age, household type, and all other variables in the Table 1. (a) In the survey year, the exchange rate was 1,000 KRW = 0.87 USD

With respect to socio-demographic factors, individuals with a high school or college education had a 34% lower likelihood of reporting MSDs compared to those with less than a middle school education (OR: 0.66, 95% CI: 0.61–0.71), while individuals with a university degree or higher had a 44% lower likelihood (OR: 0.56, 95% CI: 0.51–0.61). Regarding work-related characteristics, employed individuals had an 11% lower likelihood of reporting MSDs compared to self-employed workers (OR: 0.89, 95% CI: 0.84–0.94). Among occupational categories, skilled blue-collar workers exhibited the highest odds of MSDs, with 1.76 times the odds (OR: 1.76, 95% CI: 1.64–1.89), followed by unskilled workers with 1.35 times the odds (OR: 1.35, 95% CI: 1.24–1.47). Additionally, individuals with high job stress had 1.32 times the odds of reporting MSDs (OR: 1.32, 95% CI: 1.26–1.38), and those experiencing high physical demands had 2.15 times the odds (OR: 2.15, 95% CI: 2.06–2.25).

### Education level as a moderator between concerns about future digitalization and MSDs

**Fig. 2:**
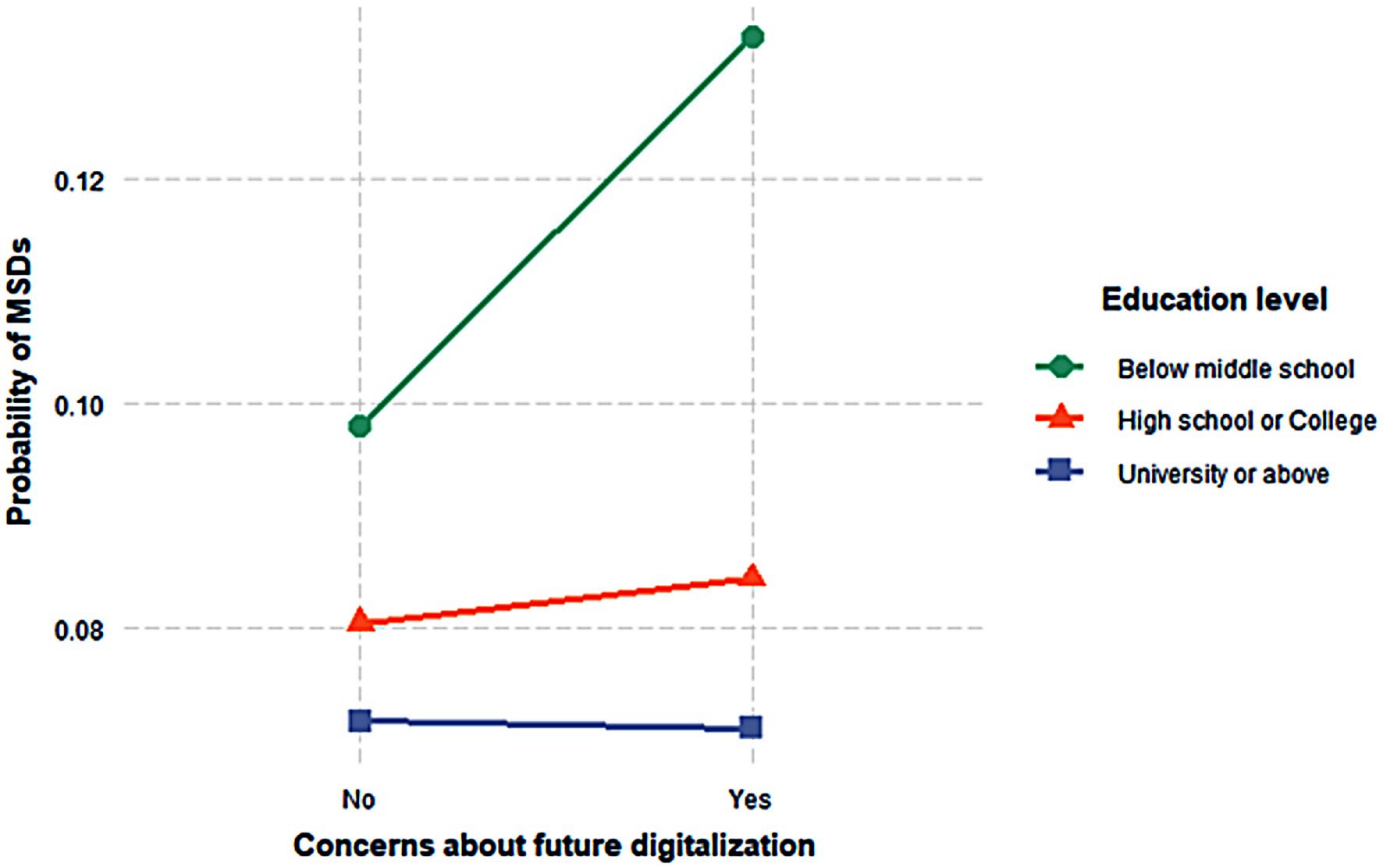
Association between concerns about future digitalization and MSDs, stratified by education level

Although potential moderating effects of socio-demographic and occupational variables—such as age, gender, and occupational category—were tested, only education level showed a statistically significant modifying effect on the association between concerns about future digitalization and MSDs, as illustrated in Fig. 2. Higher education levels had a statistically significant mitigating effect on this association. As shown in Table 3, the relationship between concerns about future digitalization and MSDs was weaker among individuals with higher levels of education. Specifically, individuals with less than a middle school education had 1.41 times the odds of reporting MSDs, whereas those with a high school or college education had a 15% lower likelihood (OR: 0.85), and those with a university degree or higher had a 29% lower likelihood (OR: 0.71), compared to the reference group.

**Table 3:** Odds ratios (ORs) for the associations between concerns about future digitalization and msds, stratified by education level (*n* = 48,604)

Model 2	Concerns about future digitalization	
	No	Yes
Education level		
Below middle school	1	1.41^‡^
High school or college	0.80^‡^	0.85 ^‡^
University or above	0.71^‡^	0.71 ^‡^

^†^*p* < 0.05, ^‡^*p* < 0.01 for main effects. *An underlined OR for the combined effects indicates that the interaction term was significant (*p* < 0.05). **Models were adjusted for socio-demographic, digitalization-related factors, and all other variables listed in Table 1

## Discussion

### Summary of key findings

This study utilized nationally representative data from the 2020 KWCS to examine the relationship between concerns about future digitalization and the likelihood of MSDs, particularly within the context of the COVID-19 pandemic. The analysis showed that concerns about future digitalization were significantly associated with MSDs, a relationship that persisted even after controlling for socioeconomic, work environment, and digitalization-related factors, making it a novel finding in the existing literature. Additionally, the study found that educational level served as an important effect modifier, with higher education levels mitigating the impact of concerns about future digitalization on MSDs.

### Key contributions of this study

Our contributions can be summarized as follows. First, while existing research on workplace digitalization had primarily focused on the physical strain related to MSDs, we addressed this gap by demonstrating that concerns about future digitalization acted as psychosocial factors in the work environment and were significantly associated with MSDs. Secondly, we found that education level moderated the association between concerns about future digitalization and MSDs, highlighting the crucial role of socioeconomic factors in this relationship. Third, we analyzed the relationship between digitalization and the likelihood of MSDs using data from the COVID-19 pandemic period. Specifically, we conducted our research by considering various factors related to the rapid acceleration of digitalization during this time, such as past adoption, current utilization, and remote work. This approach allowed us to gain a comprehensive understanding of how digital transformation impacted the physical and psychological health of workers.

### Digitalization as a new psychosocial risk factor for MSDs

Concerns about digitalization have emerged as a potential psychosocial stressor in modern workplaces, contributing to workers’ mental health challenges [27]. Although the effect size observed in our analysis is relatively modest compared to traditional risk factors such as high physical demands (OR: 2.15) or job stress (OR: 1.32), this finding remains noteworthy, as concern about digitalization constitutes a psychosocial stressor that is closely linked to ongoing structural changes in the workplace. Psychosocial stress in the workplace can influence the onset or persistence of MSDs by increasing physical strain or triggering stress responses [36].

### Evolving MSDs risks in digital work environments

Traditional physical labor has long been associated with MSDs due to repetitive movements, heavy lifting, and awkward postures, primarily affecting the lower back and shoulders [14]. In contrast, digitalized work environments, while reducing manual labor, have introduced new MSD risks linked to sedentary behavior, prolonged screen use, and static postures, often targeting the neck, shoulders, and upper limbs [10]. This shift illustrates how the primary risk factors for MSDs are evolving from purely biomechanical causes to a broader range of work environment–related factors. This is supported by our finding that concerns about future digitalization were significantly associated with MSDs (OR: 1.08; Table 2), even after adjusting for conventional risk factors. As digital transformation continues, emerging psychosocial risks—such as technostress and cognitive overload—may increasingly contribute to MSD prevalence in knowledge-based occupations [37, 38].

### Physiological mechanisms underlying psychosocial stress and MSDs

The impact of psychosocial stress on MSDs may be explained by multiple physiological and behavioral mechanisms. Stress can lead to the overactivation of the central nervous system, increased muscle tension, fewer opportunities for muscle relaxation, and impaired tissue recovery [14, 36]. Additionally, the activation of the hypothalamus-pituitary-adrenal (HPA) axis, which plays a role in the neurobiology of pain, and the secretion of pro-inflammatory cytokines, which promote pain centralization and microinflammation in soft tissues, can also occur [23, 39]. Work-related stress can also result in behavioral changes, such as altering body posture when required to complete tasks within a given time or taking fewer breaks, all of which can affect muscle function [36, 40].

### Disaggregated analysis of digitalization concerns

In our analysis, we sought to identify which specific concerns related to future digitalization were most strongly associated with the prevalence of MSDs. To do this, we deconstructed the composite measure of digitalization-related concerns into five distinct items: concern about reduced decision-making power, concern about the inability to utilize one’s skills and abilities, concern about reduced income, concern about being assigned uninteresting tasks against one’s will, and concern about changes in working hours contrary to one’s preferences. Each of these concerns was individually analyzed using a logistic regression model to assess its unique contribution to the likelihood of experiencing MSDs. The results indicated that, among the five items, only concern about reduced income was significantly associated with the likelihood of MSDs (OR: 1.07, 95% CI: 1.02–1.13; this finding is reported in the text only and is not included in any table). This finding suggests that financial worries are a particularly salient aspect of digitalization-related stress, potentially exacerbating MSDs.

This interpretation is supported by previous studies, which have consistently shown that financial stress can significantly worsen mental health conditions, such as depression [41]. This is because financial instability affects not only an individual’s economic situation but also their overall sense of security and well-being. For example, Ettman et al. (2023) found a strong correlation between financial stress and high rates of depression during crisis situations like the COVID-19 pandemic [42]. These findings align with our analysis, further reinforcing the idea that financial concerns are a particularly salient aspect of digitalization-related stress that can exacerbate physical health issues. Moreover, several studies have indicated that subjective financial hardship holds greater importance than objective debt measurements in relation to self-reported health or mental health outcomes, including depression and anxiety [43, 44]. This indicates that how individuals perceive their financial situation may have a more significant impact on their health than the actual financial metrics themselves, emphasizing the importance of addressing subjective financial concerns to mitigate health risks associated with digitalization.

By comparison, concerns about reduced decision-making power and the inability to utilize one’s skills did not show statistically significant associations with MSDs. One possible explanation is that the effects of these stressors may vary depending on occupational context [36]. For example, concerns about autonomy and skill utilization may be more salient in knowledge-based or managerial occupations, where cognitive independence plays a central role. In contrast, physically demanding jobs may be more directly affected by workload and physical demands. Since our analysis encompassed a wide range of occupations, the occupational specificity of these stressors may have been diluted or manifested differently across contexts. Future studies should consider occupation-specific models to better capture these differential effects.

### Body region–specific analysis of MSDs

In this study, MSDs were categorized into back pain, upper extremity symptoms (shoulders, neck, and arms), and lower extremity symptoms (hips, legs, knees, and feet), and follow-up analyses were conducted accordingly. The associations with upper symptoms (OR = 1.04, 95% CI: 0.99–1.09) and back pain (OR = 1.03, 95% CI: 0.98–1.08) were not statistically significant but showed marginally positive associations. In contrast, the association with lower symptoms (OR = 0.99, 95% CI: 0.93–1.05) was clearly non-significant, indicating no meaningful relationship with concerns about digitalization. These findings are consistent with previous research suggesting that psychosocial stressors may more strongly affect the upper body, possibly due to their interaction with repetitive tasks and postural tension commonly found in contemporary work environments [14, 36]. The results of these subgroup analyses are provided in Supplementary Table 1.

### Educational differences in the association between digitalization and MSDs

Our findings suggested that individuals with higher education levels were less vulnerable to MSDs associated with concerns about future digitalization compared to those with lower education levels. This result is consistent with previous studies, which have shown that individuals with higher education are less affected by the negative impacts of digitalization [45–47]. Workers with higher education levels are likely better equipped to adapt to technological innovations and therefore may experience relatively less concern about future shifts in the work environment caused by automation. Katz’s (2020) empirical estimates indicated that the number of jobs lost to automation was being balanced by the number of new jobs created, resulting in minimal impact on the labor market [45]. Frey and Osborne (2017), in their study on the susceptibility of 702 detailed occupations to computerization, predicted that computerization would predominantly displace low-skill, low-wage jobs in the near future, while high-skill, high-wage occupations were the least vulnerable to automation [46]. These results, therefore, suggested possible social exclusion, as the groups most at risk of job displacement were low-skilled, low-income workers.

### Building resilience in a digitalized workplace

**Fig. 3:**
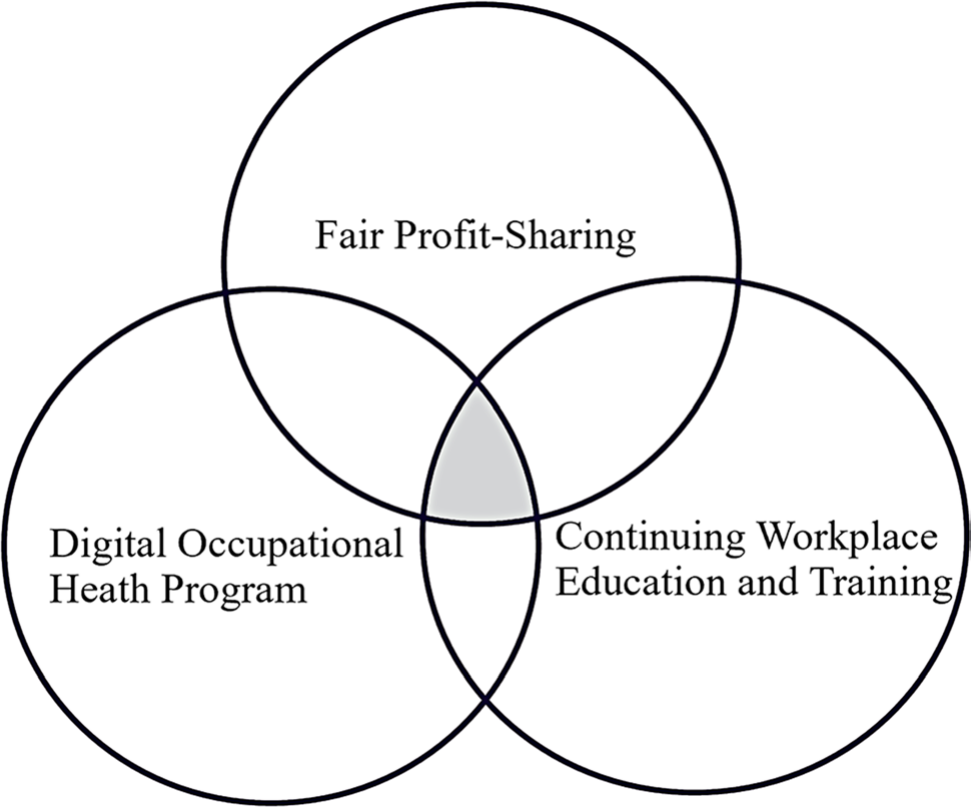
Framework for building resilient digitalization in the workplace

Resilience, the ability to overcome adversity and recover, has become increasingly critical for employees and organizations as they face new situational demands, challenges, and opportunities [48, 49]. In this evolving work environment, resilience allows workers to effectively manage stress and adapt to changes, helping them achieve better performance [50]. This capability is essential in the uncertain digital age, supporting the success and adaptability of both individuals and organizations [51]. In our study, we identified three key factors that contribute to resilience in the context of digitalization: fair profit-sharing, digital occupational health programs, and continuing workplace education and training. As shown in Fig. 3, these factors collectively form the foundation of resilience in digitalized work environments. The overlapping area in the figure represents the synergistic zone where all three conditions coexist, which we propose as the core enabling environment for workers to adapt successfully and maintain well-being amidst digital transformation.

First, we found that fears surrounding future digitalization, particularly concerns about income security, were strongly associated with MSDs. This highlights the importance of addressing not only the technical aspects of digital transformation but also the emotional and financial concerns of workers. Implementing performance-based incentives like profit-sharing can help alleviate these fears by aligning worker motivation with organizational goals [52, 53]. Such systems could provide a buffer against anxieties about income loss, enabling employees to embrace digitalization more positively.

Moreover, our findings revealed that not only did concerns about future digitalization matter, but past experiences in adopting digitalization, as well as current use of digitalization, were significantly linked to the likelihood of MSDs. Previous studies have similarly reported that while digitalization has reduced physical labor, it has introduced new risk factors, such as constant monitoring, which contribute to MSDs [54, 55]. In particular, our finding that past adoption of digitalization was closely linked to MSDs suggests that physical demands were not adequately addressed during the early stages of digitalization, and these physical strains continue to affect workers today.

To mitigate these ongoing risks, it is important to implement practical ergonomic interventions that directly address physical strain and postural stress in modern workplaces [56]. Recent studies have emphasized the effectiveness of specific measures—such as lifting equipment, lumbar support belts, and posture-corrective braces—in occupations involving prolonged standing, awkward postures, and repetitive tasks [57]. These solutions were also highly applicable to white-collar settings, where the integration of height-adjustable desks, arm supports, alternative mouse devices, and regular rest breaks was shown to reduce the risk of MSDs [58–60]. As such, redesigning the work environment in a way that maintains productivity while protecting workers’ health is crucial [61]. This includes the development and implementation of digital occupational health programs in digitalized work environments, which monitor new risk factors and protect workers’ health [62].

Lastly, our study demonstrated that higher levels of education can mitigate the health impacts associated with concerns about future digitalization, highlighting the importance of continuous workplace education and training to help workers adapt to the evolving demands of a digitalized work environment. However, previous studies found that workers with lower levels of education were significantly less likely to participate in job-related training [63]. This suggested that the workers who were most in need of retraining and skill enhancement were the least likely to receive it, further limiting their ability to adapt to the changing demands of the labor market. To address this gap, it is essential to implement tailored education and training programs specifically designed for workers with lower educational backgrounds.

### Limitations of the study

Our study is subject to several limitations. First, due to the cross-sectional design of our study, we were unable to determine the direction of the observed associations between concerns about future digitalization and MSDs. It is also possible that workers who are already experiencing MSDs may be more likely to express concerns about digitalization, rather than digitalization-related stress leading to MSDs. Therefore, reverse causality cannot be ruled out. However, prior longitudinal research has confirmed the association between psychological distress and various health outcomes, lending some support to our theoretical assumptions [64]. Second, both the exposure variable—concerns about future digitalization—and the outcome variable—MSDs—were assessed using self-reported, binary-coded responses (i.e., yes/no). These self-reported measures may be subject to recall bias and social desirability bias. In addition, the binary nature of the data limits the ability to capture variation in digitalization exposure and symptom severity. This restriction may have reduced the sensitivity of our analysis and hindered the detection of more nuanced associations. Third, we addressed missing data for the variable measuring monthly salary using the missing indicator method [65]. Approximately 4.3% of responses were marked as “no answer.” A comparison of covariate distributions between complete and missing cases revealed similar patterns, and a complete case analysis produced results consistent with those of the full sample, supporting the robustness of our findings. Finally, since this study is based on South Korean data, the findings may reflect characteristics unique to Korea’s workplace culture, digital infrastructure, and labor market structure. These contextual differences may limit the generalizability of our findings to other regions.

### Directions for future research

To advance understanding of digitalization-related MSDs, future research should employ longitudinal designs to clarify the temporal relationship between concerns about digitalization and the onset of MSDs. To enhance diagnostic precision and strengthen the validity of findings, self-reported data should be complemented with continuous or multi-level measures of digitalization exposure and clinically validated or biomechanical assessments of MSDs. Disaggregating MSDs by anatomical region (e.g., lower back, neck, upper limbs) may reveal region-specific risk factors across occupational and demographic groups. Additionally, the development of occupational health indicators tailored to digital work—such as digital workload, technostress, and remote ergonomics—could offer more policy-relevant insights. Ultimately, such research can support efforts to build worker resilience and facilitate successful adaptation to increasingly digitalized work environments.

## Conclusions

This study found that concerns about future digitalization were significantly associated with MSDs among South Korean workers, regardless of their past adoption or current use of digital technologies. Higher levels of education appeared to attenuate the association between such concerns and MSDs. Employers and policymakers are thus encouraged to recognize the psychosocial dimensions of digital transformation and incorporate them into workplace policies and ergonomic strategies. Providing targeted support for vulnerable worker groups may help reduce the burden of MSDs and promote long-term occupational health in increasingly digitalized work environments. To further advance this line of inquiry, future research should employ longitudinal designs to clarify the causal mechanisms underlying these associations. Sector-specific analyses may also help identify occupational groups particularly vulnerable to digitalization-related stress and MSDs.

## Electronic supplementary material

Below is the link to the electronic supplementary material.

Supplementary Material 1

## Data Availability

The data used in this study are available at: https://oshri.kosha.or.kr/oshri/researchField/downWorkingEnvironmentSurvey.do.

## Abbreviations

MSDs: Musculoskeletal Disorders
ICT: Information and Communication Technology
KWCS: Korean Working Conditions Survey
OR: Odds Ratio
CI: Confidence interval
SRH: Self-rated Heath
TAPI: Table assisted personal interviewing
PAPI: Pen-and-paper interviewing
