# Supplementary material for: Work-related musculoskeletal disorders and digitalization: past adoption, current utilization, and future concerns
Source: BMC Public Health. 2025 Jul 3;25:2336. doi: 10.1186/s12889-025-23466-w (PMC12225037; doi:10.1186/s12889-025-23466-w)
Supplement: Supplementary file 1 — Supplementary Material 1 [file 12889_2025_23466_MOESM1_ESM.docx]

**Supplementary Table 1.** Association between Concerns about Future Digitalization and MSDs by Body Region (n = 48,604)

|  | **Back Pain** | **Upper Symptoms** | **Lower Symptoms** |
| --- | --- | --- | --- |
|  | **Odds ratio (95% confidence interval)** | | |
| **Education level** |  |  |  |
| Below middle school | 1.00 | 1.00 | 1.00 |
| High school or College | 0.66 (0.61–0.71)^‡^ | 0.78 (0.73–0.84)^‡^ | 0.65 (0.60–0.71)^‡^ |
| University or above | 0.55 (0.50–0.61)^‡^ | 0.66 (0.60–0.72)^‡^ | 0.56 (0.51–0.63)^‡^ |
| **Employment type** |  |  |  |
| Self-employed | 1.00 | 1.00 | 1.00 |
| Employee | 0.87 (0.82–0.92)^‡^ | 0.84 (0.80–0.89)^‡^ | 0.82 (0.77–0.87)^‡^ |
| Employs others | 1.06 (0.93–1.21) | 0.89 (0.79–1.01) | 1.02 (0.89–1.16) |
| **Occupational category** |  |  |  |
| White collar | 1.00 | 1.00 | 1.00 |
| Sales and services | 1.04 (0.97–1.12) | 1.11 (1.04–1.18)^†^ | 1.11 (1.02–1.21)^†^ |
| Skilled blue collar | 1.60 (1.48–1.72)^‡^ | 1.79 (1.66–1.92)^‡^ | 1.71 (1.56–1.87)^‡^ |
| Unskilled and others | 1.24 (1.13–1.36)^‡^ | 1.40 (1.28–1.53)^‡^ | 1.39 (1.25–1.55)^‡^ |
| **Shift type** |  |  |  |
| Non-shift | 1.00 | 1.00 | 1.00 |
| Shift | 1.02 (0.94–1.12) | 1.06 (0.98–1.15) | 0.92 (0.82–1.01) |
| **Work hours (per week)** |  |  |  |
| <40 | 1.00 | 1.00 | 1.00 |
| 40–54 | 0.98 (0.92–1.05) | 1.05 (0.99–1.11) | 0.96 (0.90–1.03) |
| ≥55 | 1.26 (1.15–1.36)^‡^ | 1.48 (1.36–1.60)^‡^ | 1.24 (1.13–1.35)^‡^ |
| **Job stress** |  |  |  |
| Low | 1.00 | 1.00 | 1.00 |
| High | 1.41 (1.34–1.48)^‡^ | 1.28 (1.23–1.34)^‡^ | 1.33 (1.25–1.40)^‡^ |
| **Physical demands** |  |  |  |
| Low | 1.00 | 1.00 | 1.00 |
| High | 1.94 (1.86–2.04)^‡^ | 2.24 (2.14–2.34)^‡^ | 2.14 (2.03–2.26)^‡^ |
| **Job insecurity** |  |  |  |
| Low | 1.00 | 1.00 | 1.00 |
| High | 0.98 (0.93–1.03) | 0.97 (0.93–1.02) | 0.97 (0.92–1.02) |
| **Digitalization at Work** |  |  |  |
| **Past Adoption** |  |  |  |
| No | 1.00 | 1.00 | 1.00 |
| Yes | 1.57 (1.45–1.69)^‡^ | 1.55 (1.44–1.67)^‡^ | 1.48 (1.35–1.63)^‡^ |
| **Current utilization** |  |  |  |
| No | 1.00 | 1.00 | 1.00 |
| Yes | 1.05 (0.99–1.12) | 1.08 (1.02–1.14)^†^ | 1.02 (0.95–1.09) |
| **Remote work** |  |  |  |
| No | 1.00 | 1.00 | 1.00 |
| Yes | 1.34 (1.24–1.45)^‡^ | 1.19 (1.11–1.29)^‡^ | 1.22 (1.12–1.33)^‡^ |
| **Concerns about future digitalization** |  |  |  |
| No | 1.00 | 1.00 | 1.00 |
| Yes | 1.03 (0.98–1.08) | 1.04 (0.99–1.09) | 0.99 (0.93–1.05) |

^†^*p* < 0.05, ^‡^*p* < 0.01. a. Odds ratios were adjusted for age, family type, and all other variables in the Table 1.
